# Synthetic Sulfated Polymers Control Amyloid Aggregation of Ovine Prion Protein and Decrease Its Toxicity

**DOI:** 10.3390/polym14071478

**Published:** 2022-04-05

**Authors:** Pavel Semenyuk, Diana Evstafyeva, Vladimir Izumrudov, Vladimir Muronetz

**Affiliations:** 1 Belozersky Institute of Physico-Chemical Biology, Lomonosov Moscow State University, 119234 Moscow, Russia; evstafevadiana@gmail.com (D.E.); vimuronets@belozersky.msu.ru (V.M.); 2 Department of Chemistry, Lomonosov Moscow State University, 119234 Moscow, Russia; izumrud@belozersky.msu.ru

**Keywords:** prion protein, amyloid, polyanion, amyloidosis, artificial chaperone, polystyrene sulfonate

## Abstract

Amyloid aggregation, including aggregation and propagation of prion protein, is a key factor in numerous human diseases, so-called amyloidosis, with a very poor ability for treatment or prevention. The present work describes the effect of sulfated or sulfonated polymers (sodium dextran sulfate, polystyrene sulfonate, polyanethole sulfonate, and polyvinyl sulfate) on different stages of amyloidogenic conversion and aggregation of the prion protein, which is associated with prionopathies in humans and animals. All tested polymers turned out to induce amyloid conversion of the ovine prion protein. As suggested from molecular dynamics simulations, this effect probably arises from destabilization of the native prion protein structure by the polymers. Short polymers enhanced its further aggregation, whereas addition of high-molecular poly(styrene sulfonate) inhibited amyloid fibrils formation. According to the seeding experiments, the protein–polymer complexes formed after incubation with poly(styrene sulfonate) exhibited significantly lower amyloidogenic capacity compared with the control fibrils of the free prion protein. The cytotoxicity of soluble oligomers was completely inhibited by treatment with poly(styrene sulfonate). To summarize, sulfonated polymers are a promising platform for the formulation of a new class of anti-prion and anti-amyloidosis therapeutics.

## 1. Introduction

Amyloid fibrils formation is associated with a lot of diseases, so-called amyloidosis, including neurodegenerative diseases, such as Alzheimer’s and Parkinson’s diseases. [1,2]. Among these types of amyloidosis, APrP amyloidosis (prion disease) is of special interest since it can be transferred from one patient to another one via a misfolded form of the prion protein (PrP), a unique protein-based infectious agent. PrP can undergo conversion from a “normal” cellular form to a misfolded, so-called scrapie form, which is prone to form amyloid fibrils [3,4,5].

Preventing amyloid aggregation was considered as a promising way for treatment of diseases associated with amyloidosis. Among many suggested approaches, one should mention the use of molecular chaperones [6,7]. Thus, overproduction of Hsp70 and Hsp104 inhibited the propagation of yeast prion and huntingtin [8,9,10], and some therapeutic effect of Hsp70 has also been shown for Alzheimer’s disease [11]. However, practical use of molecular chaperones is complicated by the complexity of regulation and tuning of the chaperone system, and sometimes similar chaperones can exhibit opposite effects on amyloid aggregation [10].

We suggest the use of synthetic and natural polymers as artificial chaperones which can recognize unfolded state of the enzyme [12,13] and protect the bound protein against aggregation [14,15,16]. Furthermore, sulfated and sulfonated polymers were recently shown to be capable of disaggregation protein aggregates of different types [17]. Solubilization of glyceraldehyde-3-phosphate dehydrogenase thermal aggregates by polymers was accompanied by partial recovery of enzymatic activity. It is noteworthy that amyloid aggregates of PrP also can be disrupted by polyelectrolytes: treatment of PrP inclusion bodies by highly polymerized poly(styrene sulfonate) [17] and cationic dendrimers [18] was found to reduce amount of amyloid structures. There are also data that cationic dendrimers inhibit aggregation of beta amyloid peptide [19]. In addition, there is a large body of evidence of interaction of different amyloidogenic proteins with natural proteoglycans which contain sulfated polysaccharides [20,21,22]. Thus, heparin, heparan sulfate, and other glycosaminoglycans were shown to enhance amyloid conversion and aggregation of amyloid beta peptide [23,24], alpha-synuclein [25], tau protein [26], apolipoprotein A-I [27,28], gelsolin [29], and serum amyloid A [30]. On the other hand, sulfated aromatic polymers are capable to inhibit fibrillation of alpha-synuclein in vitro [31]. As to PrP, sulfated polysaccharides were shown to inhibit accumulation of amyloid aggregates of PrP in cell culture [32], and another sulfated polymer, polystyrene sulfonate, enhanced proteolytic degradation of amyloid fibrils of PrP [33]. In other words, charged polymers, including sulfated polysaccharides, are capable of binding prion protein and its aggregates, but their influence on amyloidogenic conversion and aggregation is not clear and requires comprehensive studies.

In the present work, we performed a comprehensive investigation of the influence of a range of sulfated and sulfonated polymers on the PrP amyloid aggregation: sodium dextran sulfate (DS), sodium polystyrene sulfonate (PSS), sodium polyanethole sulfonate (PAS), and potassium polyvinyl sulfate (PVS). The choice of the polymers was dictated by their difference in in hydrophobicity (from hydrophilic DS to relatively hydrophobic PSS), structure (aromatic PSS and PAS, aliphatic PVS, and a sulfated polysaccharide, DS), and degree of polymerization (for PSS and DS) since polyanion’s anti-aggregation efficiency, as well as influence on the protein structure, depends on all these factors. We analyzed their effect on various stages of amyloid conversions—formation of prefibrillar oligomers, fibrillation, and amyloidogenic properties—of these forms of PrP. In addition to a typical set of experimental methods, we used atomistic molecular dynamics simulations, which is a powerful approach to probe interaction between proteins and charged polymers [34,35] as well as the amyloid conversion of amyloidogenic proteins [36,37,38,39]. Finally, we tested the effect of the selected polyanions on cytotoxicity of prefibrillar oligomers, which are considered as the most toxic species of PrP [2,40] and other amyloids [41,42].

## 2. Materials and Methods

### 2.1. PrP Purification

Ovine prion protein VRQ 23–234 was expressed in *E. coli* BL21 (DE3) cell culture with the pET-22b(+) plasmid. Production and purification were performed as described in [43]. Before each experiment, lyophilized PrP was dissolved in 100 mM sodium acetate buffer, pH 4.0, and then transferred into MOPS, pH 7.5, if required, using a Sephadex G-25 column. The protein concentration was measured spectrophotometrically using A_280_^0.1%^ value of 2.6.

### 2.2. Polymers

**Scheme 1:**
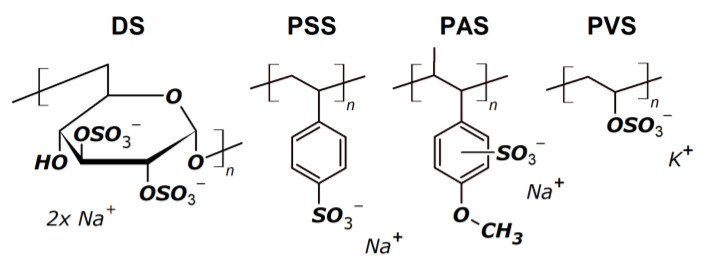
Structures of the tested polymers.

Structures of the used polymers (that all are strong polyelectrolytes and presented as polyanions) are shown in Scheme 1. Samples of sodium poly(styrene sulfonate) (PSS) with polymerization degree of 155 and 1700 (32 and 350 kDa, respectively), sodium poly(anethole sulfonate) (PAS) with approximate polymerization degree of 800 (200 kDa), sodium dextran sulfate (DS) with molecular mass of 15 kDa and 100 kDa (approximately 38 and 250 repeated units or 90 and 600 charged groups, respectively), and potassium poly(vinyl sulfate) (PVS) with approximate polymerization degree of 1100 (180 kDa) were purchased from Sigma-Aldrich (St. Louis, MO, USA).

### 2.3. PrP Aggregation Assay

PrP prefibrillar oligomers were obtained by incubation at 65 °C for 60 min in 20 mM MOPS buffer, pH 7.5 [44]. Concentration of protein was 0.5 mg/mL (1 mg/mL for cytotoxicity experiments); concentration of polyanions was 0.05–0.25 mg/mL (0.25 and 1 mM in terms of charged groups).

PrP fibrils were obtained by 3-day incubation at 37 °C in a shaker. A measure of 100 mM sodium acetate, pH 4.0, with 1 M guanidinium hydrochloride (GuHCl), 0.03% NaN_3_ was used. Protein concentration was 2 mg/mL; concentration of polyanions’ charged groups was 1.6–2.5 mg/mL (10 mM of charged groups).

Seeding experiments were performed using fibrils obtained as described above as a seed. Native PrP was transferred into 20 mM MOPS buffer, pH 7.5, with 0.03% NaN_3_, and diluted to final concentration of 0.5 mg/mL. Seed samples (fibrils formed in the presence of polyanions and reference fibrils) were added in seed to PrP ratio of 1:50 [45].

### 2.4. DLS

The size of complexes was determined by dynamic light scattering (DLS) using a ZetaSizer Nano ZS instrument (Malvern Instruments) with a scattering angle of 173°. All measurements were performed at 25 °C.

### 2.5. Thioflavin T Fluorescence Measurements

Thioflavin T (ThT) dyeing was used for analysis of amyloid properties of different PrP forms. ThT was added in 10-fold molar excess, measurements were performed 20 min later. The fluorescence was excited at 435 and the emission was measured at 450–600 nm. FluoroMax 3 (Jobin Yvon) instrument was used. In the seeding experiments, the samples were filled in the plate, a 10-fold molar excess of ThT was added, and fluorescence was measured every day during 4–5 days, with an incubation temperature of 37 °C. All experiments were repeated three times. VICTOR X Multilabel Plate Reader (PerkinElmer) was used with excitation at 435 nm and emission measurement at 480 nm.

Control experiments with mixtures of the free polymers and ThT (without the protein) were performed. For oligomerization assay, these reference values were subtracted from the values for main samples (PrP + the polymers). For fibrillization assay, spectra deconvolution was performed since background fluorescence was higher under these conditions, and therefore a simple subtraction of the controls might be incorrect.

### 2.6. Cytotoxicity Assay

SH-SY5Y cell line was obtained from Dr. Naletova, University of Catania. Cells were cultivated in flasks in DMEM (PanEco, Moscow, Russian Federation), with 10% FBS (Hyclone, Logan, UT, USA), 1 mM L-glutamate (PanEco, Moscow, Russian Federation), and a penicillin–streptomycin mixture (50 U/mL penicillin and 50 µg/mL streptomycin). Before experiments, cells were plated on 96-well plates (100 µL of cells for each well, 10^5^ cells/mL) and incubated for 24 h at 37 °C in the atmosphere of 5% CO_2_.

Aliquots (20 µL) of complexes of prion oligomers and polyelectrolytes, free prion oligomers, or free polyelectrolytes themselves were added to cells and incubated for 48 h. Cell viability was assessed with resazurin test. A measure of 20 µL of 0.15 mg/mL resazurin was added to each well. After 12 h of incubation at 37 °C, absorbance was measured using VERSAmax Tunable Microplate Reader (Molecular Devices Corporation, San Jose, CA, USA) at 570 nm for resorufin and 600 nm for resazurin. The final result was obtained by subtracting the absorbance at 600 nm from the absorbance at 570 nm.

### 2.7. Statistical Analysis

All experiments were repeated at least three times. In all figures, the averaged over independent experiments and a standard deviation are presented. For ThT fluorescence of various forms of PrP and for cytotoxicity assay, statistical significance of the difference was estimated using the Mann–Whitney test.

### 2.8. MD Simulations

The structure of PrP was retrieved from PDB database, PDB ID 1tqb (chain A). The polymer molecules were parameterized by using the RED III tools [46]. Geometry optimization of the monomers was performed in the Firefly QC package [47], which is partially based on the GAMESS (US) [48] source code. Molecular dynamics simulations were performed using GROMACS 5.1 software [49]. The GROMOS 54a7 force field was used. The simulation box contained one polymer molecule and three protein molecules; the distance between macromolecules was no less than 1 nm. Before construction of the box, the polymers molecules were relaxed for 10 ns in water solution. The length of main simulations of the protein–polymer systems in water was 100 ns with the step of 2 fs. Periodic boundary conditions and the particle mesh Ewald method for handling long-range electrostatic interaction were used. The simulations were conducted using an NPT ensemble. The temperature in the simulation box was stabilized at 300 K using velocity rescale thermostat. Pressure coupling was performed with the Berendsen algorithm [50]. The threshold distance for calculation of nonbonded contacts number was 0.35 nm for ion pairs and H-bonds and 0.45 nm for pairs of nonpolar atoms. To estimate the average distance between charged groups of the protein or the polyanions, radial distribution function was calculated for the positively charged nitrogen atoms of PrP around themselves or sulfur atoms of PSS and DS around themselves. APBS plugin for PyMOL was used to color PrP monomer according to electrostatic potential.

## 3. Results

### 3.1. Amyloid Conversion and Oligomerization of Prion Protein

**Figure 1:**
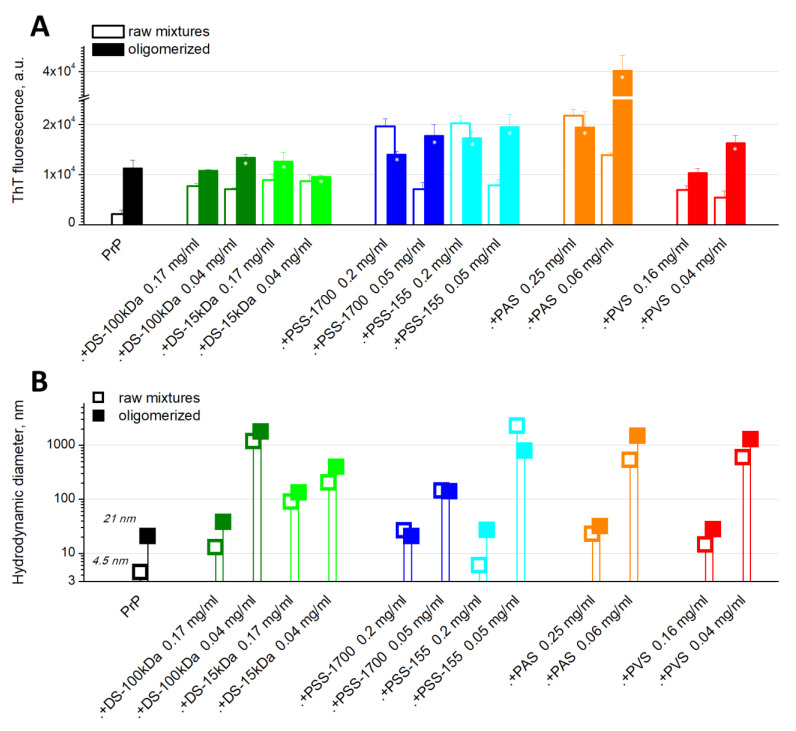
ThT fluorescence intensity ((**A**) mean ± SD, asterisks “*” mark statistically significant difference from PrP oligomers) and hydrodynamic diameter of particles (**B**) in the solution of free PrP (black bar/squares) and mixtures of PrP and the polyanions (colored bars/squares) mixed at room temperature (empty bars/squares) and after an oligomerization-inducing treatment (i.e., 1 h incubation at 65 °C) (filled bars/squares).

The study of the influence of sulfated and sulfonated polymers on amyloid aggregation of ovine PrP was started from the investigation of influence on amyloid conversion and oligomerization. All tested polymers, i.e., DS, PSS, PAS, and PVS, turned to modulate amyloid conversion, i.e., conformational transformation of native form causing formation of soluble oligomers. Indeed, PrP incubation in the presence of the polymers under natural conditions led to a significant increase in ThT fluorescence (Figure 1A, empty bars). This was a concentration-dependent effect; the most pronounced increase was observed in case of aromatic polymers, namely PSS and PAS, agreeing with the data on the effect of such polymers on the globular protein structure [16,51]. However, further oligomerization-inducing treatment to obtain prefibrillar oligomers results in only a small increase in ThT fluorescence in case of excess of the polymers but more pronounced in case of lower concentration of the polymer, whereas free PrP formed conventional soluble oligomers with an increase in ThT fluorescence (Figure 1A, black bar).

On the other hand, the size of the complexes did not increase during the oligomerization-inducing treatment (a significant difference between raw mixtures and treated samples was observed only for PSS-155), suggesting that further oligomerization/aggregation does not occur (Figure 1B). Relatively large complexes were formed in all cases immediately after mixing the PrP and polymers. The size of these complexes seems to depend generally on the polymer and its concentration: at excess of the polyanion, smaller complexes were formed compared to a lower concentration of the polymer that is usual for protein–polyelectrolyte systems. In the case of excess of longer chains, the size of complexes was similar to the size of PrP oligomers.

Summarizing these results, one can conclude that all tested polymers induced amyloid-like conversion of PrP. A higher concentration of the polymers (1 mM in terms of charged groups, i.e., 0.16–0.25 mg/mL) was enough for a complete conversion. At 0.25 mM of the polymers’ charged groups (0.4–0.6 mg/mL), conversion was not complete, and oligomerization-inducing treatment caused further conversion/aggregation, which arises from intra-complex interaction since the complexes do not increase in size due to such treatment. The higher values of ThT fluorescence of the samples with PSS and PAS seem to be associated with higher influence of these polymers on the protein structure. In addition, the values of ThT fluorescence were relatively similar to that for PrP oligomers instead of fibrils, indicating that amount of cross-beta structures was relatively small.

### 3.2. Modeling of Protein–Polymer Interaction

**Figure 2:**
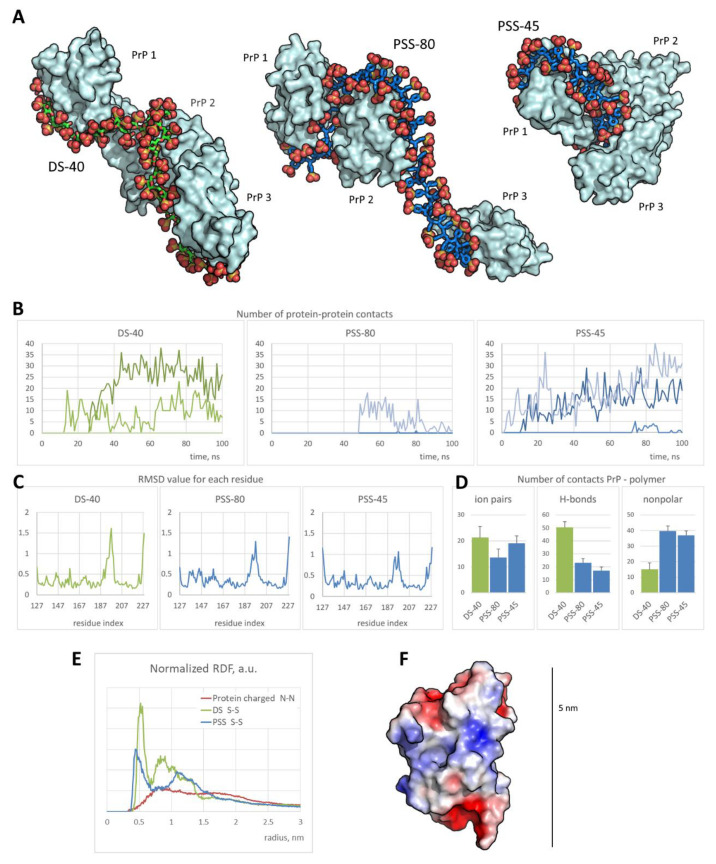
Results of MD simulations of PrP and polyanions interaction. (**A**) Final complexes formed after 100 ns simulations. Protein molecules are shown in surface representation and colored in pale cyan; DS and PSS molecules are shown in stick representation and colored in green and blue, respectively; sulfate/sulfonate groups in DS and PSS are shown in spheres representation. (**B**) The number of protein–protein nonbonded contacts between PrP molecules. (**C**) RMSD per residues profiles calculated for heavy atoms of PrP and averaged among PrP molecules for last 10 ns. (**D**) The number of contacts (mean ± SD) between protein and polymer. (**E**) Radial distribution function for the positively charged groups of PrP around themselves and sulfonate/sulfate groups of PSS and DS around themselves. (**F**) General view on PrP monomer colored according to electrostatic potential (blue for positive and red for negative; determined using APBS).

To probe the mechanism of the action of charged polymers on PrP, we used atomistic molecular dynamics simulations. The system contained one charged chain of the polymer and three PrP molecules, which correspond to excess of protein molecules used in the abovementioned experiments (approximately 30–140 charged groups of the polyanions to three protein molecules). PrP molecules efficiently interacted with the polymer chain during the simulation. Three different polyanions were studied: PSS-80 and DS-40, which contain 80 sulfate groups since 2 hydroxyl groups of each DS repeat unit were sulfated, and PSS-45 with only 45 charged groups. In all cases, the polymer chain was able to bind all three PrP molecules (Figure 2A). Such simultaneous binding of three protein molecules with polymer chain caused a convergence of them to each other that consequently resulted in the formation of protein–protein contacts in case of DS and shorter PSS chain (Figure 2B). In the case of PSS-80, the number of protein–protein nonbonded contacts was very small, and the few formed contacts were not stable over the simulation. In other words, binding with DS induces protein–protein interaction, in contrast to PSS of the same polymerization degree, but with similarity to the twice-shorter PSS chain.

The effect of the bound polymers on the PrP structure was similar (Figure 2C): in all cases, rearrangement of the loop between B and C helices (residues 187–205) was observed. This region might be important for PrP amyloidogenic conversion [36]. However, the binding of PrP with PSS and DS differs in details of the formed contacts (Figure 2D). Although both polymers form numerous ion pairs with the protein, complexes of PrP and DS are also stabilized by H-bonds, whereas more hydrophobic PSS forms hydrophobic contacts with PrP. This difference might be associated with a higher level of PrP amyloid conversion caused by PSS binding compared to DS binding demonstrated in the previous section (Figure 1A).

In addition, average distance between sulfate/sulfonate groups of the polyanions was estimated from radial distribution functions of these groups around themselves (Figure 2E). The minimal occupied distance (i.e., the first peak in RDF profile) for both polyanions (0.52 and 0.44 for DS and PSS, respectively) was smaller than the typical distance between positively charged groups on the protein surface, suggesting a bad fit of charge patterns of both polyanions to those of PrP. On the other hand, second peaks on RDF profiles of both polymers are closer to a typical distance between positively charged groups of PrP. These peaks roughly correspond to a “width” of the polyanion chain and look to fit to the size of positively charged patches on the PrP surface (Figure 2F), which form a kind of belt on the PrP monomer and efficiently bind polyanionic chains. Based on the typical distances between charged groups, one can suggest that the DS chain is slightly thinner and might be a better fit to the positively charged regions of the PrP surface.

### 3.3. Formation of Fibrils

**Figure 3:**
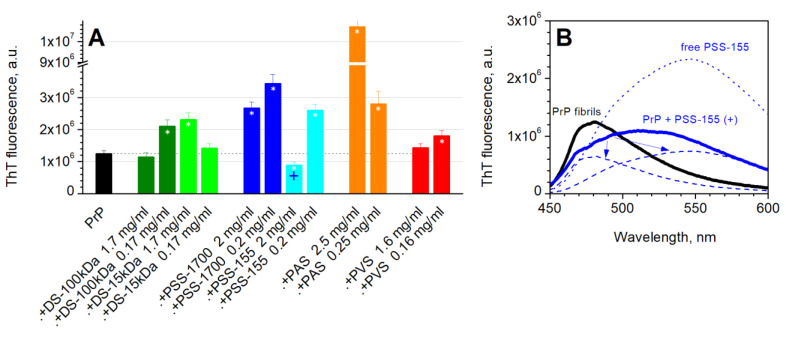
(**A**) ThT fluorescence in the presence of control PrP amyloid fibrils (black bar) and complexes formed in mixtures of native PrP and the polymers under the same fibrillization-inducing conditions. Mean ± SD values are shown; white asterisks “*” mark statistically significant difference from PrP fibrils. (**B**) Deconvolution of the fluorescence peak corresponding to PrP and 10 mM PSS-155 mixture (i.e., the bar marked with “+” in A panel): control fibrils (black line), the complexes formed under the same conditions in the presence of PSS-155 (thick blue line), free PSS-155 (thin dotted blue line), and the result of deconvolution (thin dashed blue lines).

Then, we studied the influence of the sulfated polymers on the formation of amyloid fibrils. For this, native monomeric PrP in free form and in the presence of the polymers was incubated at 37 °C for 3 days in sodium acetate buffer, pH 4.0, with 1 M guanidinium hydrochloride. Long chains of DS (100 kDa) exhibited no effect on the ThT fluorescence at 1.7 mg/mL (10 mM of charged groups) and even led to enhanced fibrillization at 0.17 mg/mL (Figure 3A). A similar picture was observed for PVS. For aromatic polymers, the effect was completely different. PAS dramatically enhanced the formation of amyloid fibrils. PSS-1700 also enhanced fibrillization. On the contrary, shorter chains of PSS, namely PSS-155, demonstrated a partial inhibition of amyloid aggregation. It should be noted that Figure 3A represents data without correction for probable polymer-driven ThT fluorescence in absence of the protein that takes place for PSS. According to the corrected data (we used peak deconvolution since the peaks of ThT fluorescence in presence of PSS itself and amyloid fibrils themselves overlap and therefore a simple subtraction can give artificial result), amyloid aggregation was inhibited for 48% by PSS-155 at 2 mg/mL (10 mM of charged groups, Figure 3B).

### 3.4. Amyloidogenic Capacity of Fibrils (Seeding)

**Figure 4:**
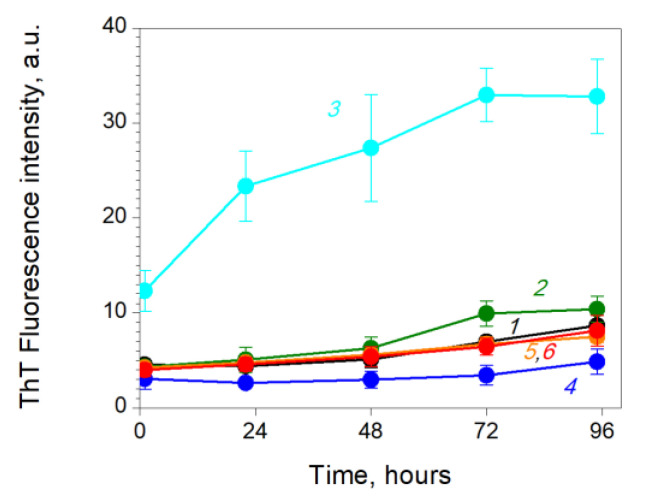
Seeding of native PrP solution with PrP fibrils (curve **1**, black) and the complexes formed in the same conditions in the presence of the polyanions: DS-100kDa (**2**), PSS-155 (**3**), PSS-1700 (**4**), PAS (**5**), and PVS (**6**). MOPS 20 mM, pH 7.5. Values averaged over independent samples ± SD are shown.

Amyloidogenic capacity of the formed complexes was examined in seeding experiments. Small portions of the complexes or the control fibrils obtained by incubation in the absence of the polymers were added to native PrP in 20 mM MOPS, pH 7.5, in ratio of 1:50. Surprisingly, the complexes of PrP with PSS-155, which contain less amyloid fibrils according to the abovementioned data, were significantly more amyloidogenic compared with the control fibrils (Figure 4). A similar but less pronounced effect was observed for DS, while PAS and PVS did not influence aggregation. However, amyloid aggregation of PrP, induced by the seeded complexes formed in the presence of the longest PSS chains, namely PSS-1700, was significantly less efficient than that induced by seeds of control fibrils. Note that concentration of the polymers in the final sample was 50 times lower than in experiments with influence of free polymers on PrP oligomerization, since they were contained in only a small seeding volume; therefore, the observed effect should be attributed to amyloidogenic capacity of the complexes instead of the action of free polyanions, which probably exist in the seeding aliquots. In other words, the fibrillization of PrP in the presence of long PSS chains resulted in the formation of low-amyloidogenic complexes.

### 3.5. Cytotoxicity of PrP Oligomers

**Figure 5:**
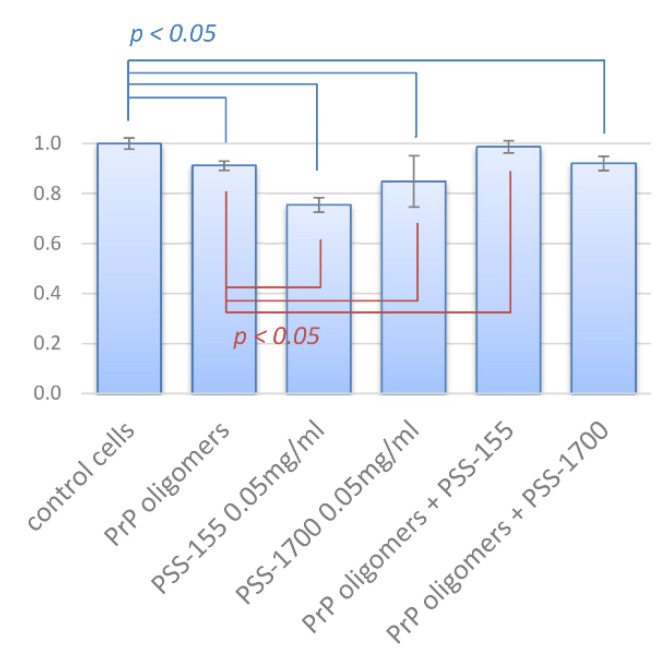
Neuroblastoma cells viability in the presence of PrP oligomers and PrP oligomers incubated with PSS-155 or PSS-1700 as well as PSS themselves. A statistically significant difference with control cells and control cells incubated with PrP oligomers (*p* < 0.05) is marked with blue and red brackets, respectively.

Toxicity of prion oligomers was tested on neuroblastoma cell line SH-SY5Y by resazurin test. Although the addition of free oligomers significantly decreased cell viability (which agrees well with data from literature), no cytotoxicity of the complexes with PSS-155 was observed (Figure 5). Decrease in toxicity was statistically significant (*p* < 0.05). In the case of longer chains of PSS, namely PSS-1700, a significant effect was not observed, that can be associated with toxic action of the polyanion itself, as well as with the lack of fibrillization inhibition by PSS-1700 (Figure 3). However, we can conclude that PSS-155 neutralizes the toxic effect of oligomers on neuroblastoma cells (Figure 5). Interestingly, the toxic effect of free PSS-155, which is higher than the toxic effect of PSS-1700, was also reduced due to complexation with PrP.

## 4. Discussion and Conclusion Remarks

**Figure 6:**
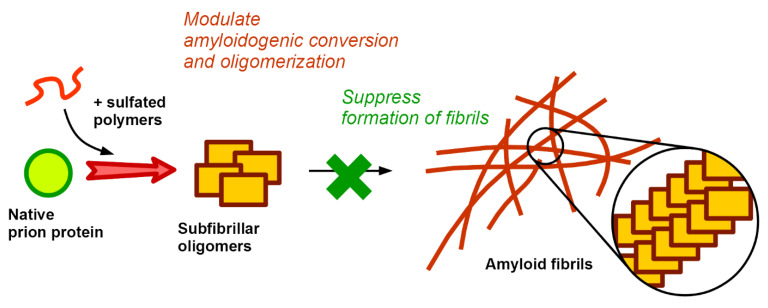
The scheme of the anti-amyloid action of sulfated polymers.

In summary, sulfated/sulfonated polymers influence amyloid aggregation of ovine PrP. A set of different polymers were tested: sulfated polysaccharides (DS), aromatic polymers (PSS and PAS), and a sulfated aliphatic polymer (PVS). Based on the obtained results, one can conclude that all tested sulfated polymers induce amyloid-like conversion of PrP. The effect of longer charged chains, whose influence on the structure of the bound enzyme is known to be smaller [51], was less pronounced. However, further aggregation under oligomerization-inducing conditions and, importantly, under fibrillization-inducing conditions, can be inhibited by poly(styrene sulfonate). Furthermore, the stable complexes formed in presence of long PSS chains have low amyloidogenicity. In other words, despite enhancement of amyloid conversion, further amyloid-like aggregation and prion propagation can be inhibited by addition of sulfated polymers (Figure 6).

The obtained results provide a prospective platform for the creation of a new class of anti-prion agents. Anti-amyloid action of sulfated aromatic polymers was recently demonstrated for another amyloidogenic protein, human alpha-synuclein, which is a key protein involved in the development of Parkinson’s disease [31]. PrP is also important since is associated with a set of amyloidosis in humans and cattle, serving as a unique protein-based inflammatory agent (prion). From this point of view, the most prospective finding in our research is that PrP aggregation can be inhibited with formation of stable complexes with low amyloidogenic capacity (i.e., capacity for seeding of native prion protein with amyloidogenic form); therefore, prion propagation can be inhibited. Of note is that complexes formed in the presence of PSS were shown to have reduced cytotoxicity compared to oligomers of the free PrP.

The obtained data suggest that two main factors can influence PrP behavior in the presence of charged polymers: destabilization/stabilization of the protein structure and the control of protein–protein interactions. According to the experiments, all tested polymers induced amyloidogenic transformation of PrP. This effect was the most pronounced for aromatic polymers: PSS and PAS. Both these polymers were recently shown to inhibit amyloid aggregation of another amyloidogenic protein, alpha-synuclein [31]. However, only PSS exhibited anti-amyloid action towards PrP, whereas PAS significantly enhanced PrP fibrillization. This difference might arise from the protein-specific details in the interaction. Alternatively, one can note that the anti-prion action of PSS strongly depends on the polymerization degree of the polymer, and we could not claim that another fraction of PAS has the same pro-fibrillization effect as the tested one. Furthermore, the effect of the degree of polymerization of PSS on the effect on PrP aggregation is complicated: though two fractions (PSS-1700 and PSS-155) similarly induced amyloid-like conversion of native PrP, only shorter chains inhibited fibrillization, whereas longer chains enhanced aggregation level. In the contrast, the formed complexes in the case of PSS-155 were extremely amyloidogenic in seeding experiments, whereas the complexes formed in the case of PSS-1700 were significantly less amyloidogenic compared with control fibrils. Such a high amyloidogenicity of the complexes formed under fibrillization-inducing treatment in the presence of PSS-155 might be associated with anti-fibrillization activity of this polymer: in the complex, PrP is stabilized in a state which is not prone to form fibrils but is able to interact with a cellular (normal) form of PrP. This behavior can be linked with the idea that formation of fibrils is an anti-amyloidosis, protective pathway, since fibrils are less toxic than prefibrillar oligomers. In other words, inhibition of PrP fibrillization by the shorter chain of PSS might result in the formation of toxic complexes in contrast to enhancing fibrillization by longer chains of PSS, which results in formation of large but non-amyloidogenic (and nontoxic) complexes.

Finally, the level of amyloid conversion induced by DS was lower compared with the action of PSS. Thereby, one can mention that kosmotropes such as sulfate anions might promote amyloid polymerization [52]. Although the concentration of the anionic groups tested in the present research was small, a local effect inside the complex might be assumed. On the other hand, although DS and PSS have similar anionic groups, the structure and nature of these polymers is different: a backbone of PSS is much more hydrophobic compared with that of DS. Results of our molecular modeling demonstrated that PSS forms hydrophobic interaction with PrP, whereas DS does not. Unfortunately, such modeling is not enough to predict the effect of the polymer on the PrP. However, taken together with experimental data, it can provide new information about the binding and possible actions, including the effect on protein–protein interactions. It is of interest as a model of biological processes, since dextran sulfate is similar to natural sulfated polysaccharides, such as heparan sulfate; therefore, it might help in elucidating the role of these polymers in the progression of amyloidosis. Thus, heparin, heparan sulfate, and other glycosaminoglycans can modulate fibrillization of amyloid beta peptide and seem to play an important role in Alzheimer’s disease progression [53,54]. A similar pro-amyloid effect of sulfated polysaccharides was demonstrated for alpha-synuclein [25], tau protein [26], and other amyloidogenic proteins (see more examples in Introduction). On the other hand, anti-prion activity of a range of sulfated polysaccharides was shown in [32]; although, our results do not demonstrate a similar effect of DS. This might arise from a strong dependence of the effect from the properties of particular polymer (including degree of polymerization) and tested conditions: in the listed studies, different sulfated polysaccharides exhibited different effects. Alternatively, the effect of the sulfated glycosaminoglycans on different stages of a process as complex as amyloid-like aggregation can be different, as was the case for PSS in the present work. Furthermore, in living cells, the system might be complicated (at least for long chains of polysaccharides) by the molecular crowding effect, which was shown to effect protein aggregation, including amyloid-like aggregation [55,56]. As a result, an overall effect on fibrils accumulation in cells is difficult to predict. A comprehensive study of different sulfated polysaccharides should be a focus for future study.

## Data Availability

Data available on request.
